# Investigating the efficacy of the application of tension-reducing suture technique in maxillofacial plastic and cosmetic surgery

**DOI:** 10.3389/fsurg.2025.1669865

**Published:** 2025-10-10

**Authors:** Yang Du, Jiang N. Yang, Zhi G. Liao, De Y. Fu

**Affiliations:** 1 Department of General Surgery, Clinical Medical College, Yangzhou University, Yangzhou, Jiangsu, China; 2 Department of Emergency Surgery, Changsha Economic Development Zone Hospital, Changsha, Hunan, China; 3 Department of Basic Medicine, School of Medicine, Hunan Normal University, Changsha, Hunan, China

**Keywords:** tension-reducing suture, maxillofacial plastic surgery, cosmetic suturing, scar width, Vancouver scar scale (VSS), facial trauma

## Abstract

**Objective:**

This study aimed to explore the application effect of tension-reducing suture technique in maxillofacial plastic surgery.

**Methods:**

The clinical data of 80 patients with maxillofacial trauma who underwent plastic surgery and cosmetic suture in the outpatient operating room of our hospital from March 2022 to March 2024 were retrospectively analyzed. The experimental group used tension-reducing suture technology, while the control group used traditional suture technology. The patients were divided into two groups according to the type of suture, with 40 cases in each group. The two groups were compared in terms of perioperative indicators, complication rate, total satisfaction rate, scar width, and scar quality indicators.

**Results:**

The perioperative indexes, complication rates, and total satisfaction rate of the experimental group after surgery were significantly different from those of the control group (*P* < 0.05). The VSS scores of the experimental group were also significantly lower at 1, 3, 6, 12 months after surgery (*P* < 0.05).

**Conclusion:**

The tension-relieving suture technique has significant advantages in maxillofacial plastic surgery. It can effectively reduce surgical complications, reduce scar width, improve patients' scar appearance score, improve the total quality of life score and treatment satisfaction, and is worthy of clinical promotion.

## Introduction

As an important functional and aesthetic area of the human body, the face is at high risk of exposure to various external force traumas, which are common in traffic accidents, sports injuries, falls and other events (1). Facial soft tissue trauma not only affects the patient's physiological function, but also has a significant impact on appearance and mental health, such as social disorders and psychological stress reactions (2, 3). Although traditional emergency debridement and suturing can effectively stop bleeding, close the wound and prevent infection, it often ignores the structural characteristics and aesthetic needs of facial tissue, resulting in obvious postoperative scars and uneven skin tension, affecting the patient's facial appearance and quality of life (4).

In recent years, with the continuous development of plastic and cosmetic surgery technology, cosmetic suturing technology has gradually been applied and promoted in the treatment of emergency facial trauma. Cosmetic suturing not only emphasizes aseptic operation and tissue alignment, but also pays more attention to tension-reducing sutures, neat edge alignment and matching of skin texture trends. While ensuring the recovery of wound function, it minimizes scar formation and improves patient postoperative satisfaction and facial aesthetics (5, 6). This technology is particularly suitable for areas with high requirements for appearance, such as maxillofacial area, periocular area, and periorbital area (7–9).

Domestic and foreign studies have shown that emergency cosmetic suture has obvious advantages in reducing complications, reducing scar hyperplasia rate and improving patient satisfaction. Therefore, on the basis of following the principles of plastic surgery, the rational use of cosmetic suture for emergency facial trauma treatment has become an important direction of cross-integration between modern emergency medicine and cosmetic plastic surgery. This study explored the application effect of tension-reducing suture technology in maxillofacial plastic surgery and compared and analyzed related observation indicators to prove its practicality in clinical practice.

## Materials and methods

### General information

The clinical data of 80 patients who underwent maxillofacial cosmetic or reconstructive surgery in our outpatient department between March 2022 and March 2024 were retrospectively analyzed. This study was approved by the Medical Ethics Committee of our hospital (The Medical Ethics Committee of Changsha Economic Development Hospital: No.JKYY-SOP-009), and all participants provided written informed consent for the use and publication of their images in this study.

### Inclusion and exclusion criteria

#### The inclusion criteria of this study are as follows

Patients who plan to undergo maxillofacial cosmetic surgery and have high aesthetic expectations for the postoperative incision recovery effect. Patients aged over 18 years old, patients and their families have signed informed consent documents, and can understand and cooperate with postoperative follow-up and evaluation. Patients with fresh facial soft tissue injuries, in principle, can be sutured cosmetically, with neat wounds, light contamination, controllable bleeding, and clear consciousness.

#### Exclusion criteria were as follows

Patients with severe cardiovascular and cerebrovascular diseases or liver and kidney dysfunction; individuals with abnormal coagulation function; those with large-area facial trauma accompanied by deep tissue damage or open fractures; special maxillofacial deformity cases requiring complex reconstructive surgery; those with mental illness, cognitive impairment or poor postoperative compliance; those who have recently received facial-related treatments or interventions that affect their judgment of the effects; those with excessively high cosmetic expectations or those who are unable to cooperate.

### Surgical method

#### Experimental group (layer-by-layer tension-relieving suture technique)

**Figure 1:**
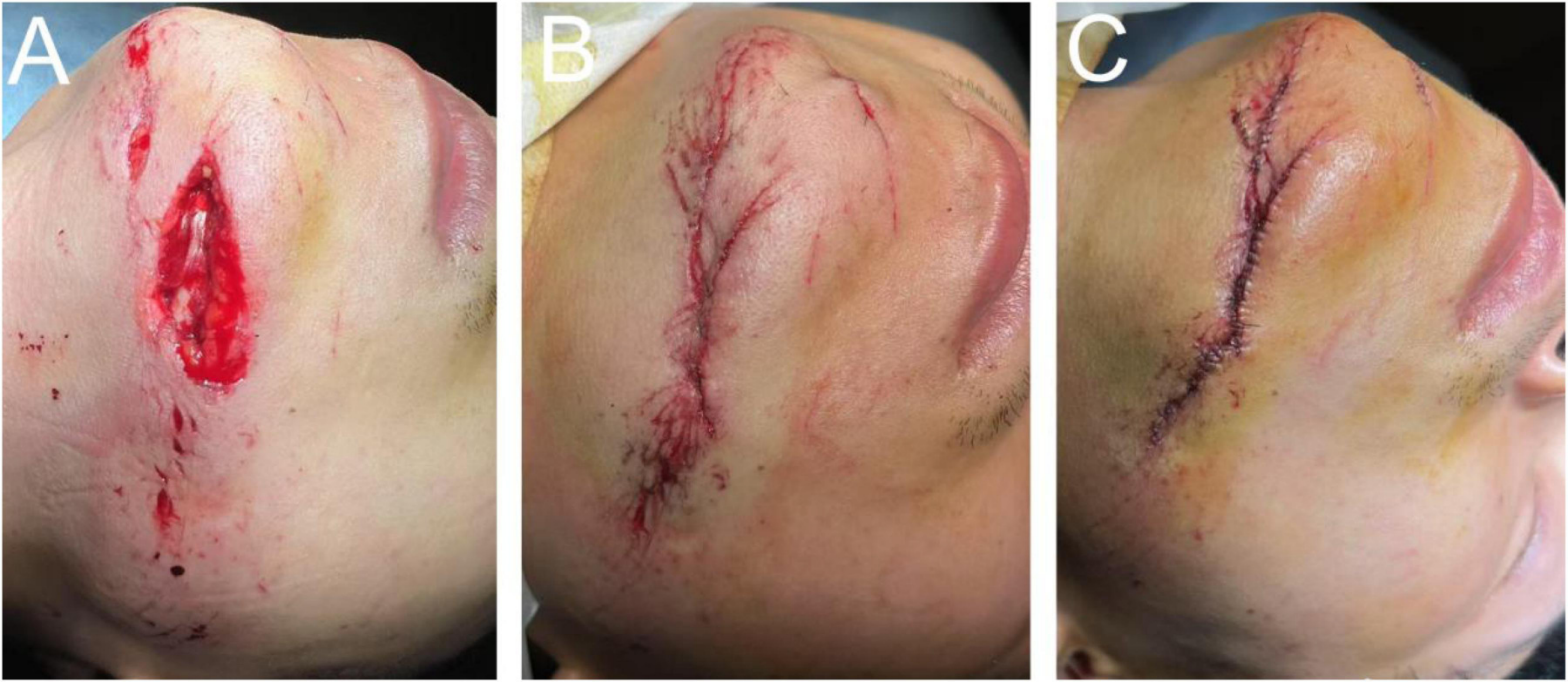
Tension-reducing suturing technique in maxillofacial reconstructive surgery. **(A)** Muscle layer suturing. **(B)** Subcutaneous tissue suturing. **(C)** Epidermal suturing.

Cosmetic suturing is guided by the anatomical and aesthetic principles of plastic surgery, emphasizing aseptic operation, tissue protection, tension-relieving suture and scar minimization. It is a high-demand but ideal suturing technique in the treatment of emergency facial trauma (10, 11). Its operation process includes the following key steps: (1) Preoperative preparation and debridement: The patient is placed in a supine position, the facial wound is fully exposed, and 0.9% saline and 3% hydrogen peroxide solution are used to alternately rinse. Multiple rinses are used to completely remove all contaminants, inactivated tissues and blood clots. For foreign bodies embedded in the lesion, micro-operation tools are selected with the help of microsurgery equipment or high-magnification magnification devices to implement precise stripping techniques. During the operation, the principle of minimally invasive surgery is strictly followed to protect the surrounding healthy tissues to the greatest extent. Afterwards, a sterile aperture drape was applied to isolate the surgical field, covering all non-operative facial areas, supplemented by triple disinfection with 0.45%−0.55% povidone-iodine, adhering to standard maxillofacial surgical protocols (12, 13). (2) Hemostasis and neurovascular protection: During the operation, check for neurovascular damage. If there is arteriovenous rupture, electrocoagulation or ligation can be used to stop bleeding. The face has a rich blood supply, and viable tissue should be preserved as much as possible. If necessary, imaging can be combined to evaluate foreign bodies and deep structures (14). (3) Layered suturing principle: Follow the principle of “no dead space, layered suture, and tension-relieving suture” and suture layer by layer from the muscle layer, subcutaneous tissue to the dermis and epidermis. The conventionally recommended sutures are as follows. ① Muscle layer: 5-0 absorbable suture, usually continuous or interrupted suture to ensure precise alignment. If there is a defect in muscle and soft tissue, patch repair can be used to restore the integrity of the structure (Figure 1A). ② Subcutaneous tissue: The needle insertion angle should be as parallel to the subcutaneous tissue plane as possible to avoid suturing too tightly and compressing the tissue, affecting its blood supply and activity; at the same time, the suture length needs to be adjusted according to the thickness of the tissue. Usually, the span of a single needle is controlled at 0.5–1 cm to ensure effective elimination of the cavity. During the suturing process, toothless forceps are used to gently pull the fat tissue around the wound toward the center so that the subcutaneous tissue can be smoothly, evenly and tension-freely fitted. It is recommended to use 5-0 absorbable sutures or 6-0 absorbable sutures for tension-reducing sutures during this operation. This series of delicate operations aims to eliminate ineffective cavities and provide stable support for the skin wound edge, thereby promoting its optimal tension-free adhesion and healing, and significantly reducing the risk of complications such as seroma, hematoma or wound dehiscence (Figure 1B). ③ Epidermis: 7-0 nylon or Prolene non-absorbable sutures are interrupted, with a needle length of 3−5 mm and a needle hole 2–3 mm from the wound edge; the needle length can be appropriately reduced for special areas such as the eyelid margin to ensure precise alignment (15). The suturing process should be gradually advanced from the midpoint of the incision to both sides, keeping the skin flat and the edges symmetrical. Apply moderate force when tying the knot to ensure that the skin fits without eversion or inversion. By adjusting the suture spacing on both sides of the skin edge, skin tension can be effectively reduced, thereby reducing the risk of wound dehiscence (Figure 1C). (4) Defect repair and pressure dressing: For larger skin defects, V-Y advancement, local flap transfer or free skin grafting can be used for repair according to the size and location of the defect. After surgery, 3 M suture-free tape is used to reduce tension on the wound edge, and the wound is covered with absorbent dressings and moderate pressure bandages are applied to reduce bleeding and tension (16). (5) Postoperative management and follow-up: Antibiotics are routinely given after surgery to prevent infection. Instruct patients to return for a follow-up visit 24 h after surgery, change the wound dressing once every 2 days, and remove the stitches after 5–7 days. Doctors need to instruct patients to clean the wound scientifically, observe signs of infection such as redness, swelling and exudation, and avoid sun exposure to reduce pigmentation and scar formation (17, 18).

This image illustrates the application of the layered tension-reducing suturing technique in a representative patient from the experimental group (e.g., a 24-year-old male patient with a 7.2 cm laceration on the left lower jaw region of the face). The photograph was taken under sterile conditions during surgery, with the patient's name anonymized for protection. The goal of preoperative and perioperative management is to ensure a sterile surgical environment, clean wound preparation, and adequate tissue protection, thereby providing optimal conditions for subsequent tension-reducing suturing techniques. For details, refer to Key Steps 1 and 2 in the surgical procedure. All surgical photographs were taken under strict sterile conditions, with an aperture drape isolating the wound to minimize contamination and background interference. The drape was adjusted during photography to clearly visualize the suturing process, but sterility was maintained throughout.

Figure 1A: Muscle layer suturing. Shows precise alignment and closure of the muscle defect using 5-0 absorbable suture in a continuous pattern, with patch repair if required. Note that electrocoagulation of small vessels is performed prior to suturing to maintain tissue viability. The pre-suture (left) and post-suture (right) views demonstrate reduced tension to prevent dead space. Figure 1B: Subcutaneous Tissue Suturing. Depicts the use of 5-0 or 6-0 absorbable suture material, with needle insertion parallel to the tissue plane at 0.5–1 cm intervals. Non-toothed forceps are employed for central approximation of adipose tissue, ensuring tension-free apposition. Arrows indicate suture knots and eliminated gaps, emphasizing perioperative management to prevent hematoma formation (e.g., gentle handling to preserve blood supply). Figure 1C: Epidermal Suturing. Illustrates interrupted suturing with 7-0 nylon suture, needle length 3–5 mm, placed 2–3 mm from wound margins. Advancing from the incision midpoint toward both ends ensures symmetrical edges without everting. The post-suturing view demonstrates tension reduction by adjusting spacing and provides additional support using 3M tape in perioperative care.

#### Control group

The traditional suturing method mechanically follows a layered sequence from deep to shallow (thick sutures and large-span suturing). Although it can quickly close the wound, it easily leads to poor healing and scar risk due to the neglect of precise tension regulation, differentiated apposition of tissue layers and dead space control. Its operation process includes the following key steps: (1) Preoperative Preparation: Similar to the experimental group, use saline and hydrogen peroxide irrigation, but minimally invasive instruments may not be emphasized. (2) Debridement and Hemostasis: Routinely examine neurovascular structures, using electrocoagulation or ligation for hemostasis, but without special emphasis on preserving viable tissue. (3) Layered Suturing: Proceed from deep to superficial layers using thicker sutures (e.g., 4-0 or 5-0 non-absorbable), with wide-spaced sutures (1–2 cm intervals). Do not emphasize tension relief or dead space elimination. (4) Postoperative Management: Administer routine antibiotics and perform wound dressings, but do not use 3M tension-free tape.

#### Observation indicators

Perioperative indicators: The length of surgical incision, operation time, duration of postoperative edema, wound healing time, and treatment costs of the two groups of patients were recorded, and the corresponding data were counted and statistically analyzed.

Surgical complications: The occurrence of postoperative complications in both groups of patients, including infection, hematoma, knot reaction, dead space formation and wound dehiscence, was recorded and the incidence was calculated.

Total satisfaction rate of treatment: After treatment, the patients were evaluated using the visual analogue scale. 6 points or below indicated dissatisfaction, 7–9 points indicated relatively satisfied, and 10 points indicated very satisfied. The total satisfaction rate was the sum of the very satisfied rate and the relatively satisfied rate.

Scar width: Using a high-precision measuring tool, the width of the widest part of the scar was measured at 1, 3, 6 and 12 months postoperatively.

Vancouver Scar Scale (VSS): The VSS scale was used to evaluate scar condition at 1, 3, 6 and 12 months after surgery. This scale is used to evaluate scar characteristics, including color, thickness, and vascularity, with a total score range of 0–15. The lower the score, the less obvious the scar (19).

The Vancouver Scar Scale (VSS) was originally developed by Sullivan et al. in 1990 as a clinical tool to assess scar quality, including vascularity, pigmentation, pliability, and height. The scale is in the public domain and has been widely adopted in clinical and academic settings without restriction. No formal permission is required for its use in non-commercial clinical research (20).

#### Data analysis method

Data were analyzed using SPSS version 25.0 (IBM Corp., Armonk, NY, USA). Continuous variables (e.g., scar width and Vancouver Scar Scale [VSS] scores) were presented as mean ± standard deviation (SD). Normality was assessed using the Shapiro–Wilk test, and homogeneity of variance using Levene's test. For data meeting both assumptions, intergroup comparisons (experimental vs. control) were performed with independent-samples *t*-tests; otherwise, the Mann–Whitney *U* test was applied. Categorical variables (e.g., complication rates, satisfaction rates) were expressed as frequencies (percentages) and compared using chi-square tests or Fisher's exact test when expected counts were <5. All tests were two-tailed, with *P* < 0.05 considered statistically significant. For repeated evaluations across multiple time points (1, 3, 6, and 12 months), individual *P* values were reported for each time point. Sensitivity analyses were performed by reanalyzing data with nonparametric tests and excluding outliers (values >3 SD from the mean). No missing data were identified; therefore, imputation was not required.

## Results

Baseline Characteristics: The experimental group (*n* = 40; 10 males, 30 females) underwent a layer-by-layer tension-reducing cosmetic suture technique. The age range of the subjects was 19 to 54 years (mean ± standard deviation (SD): 33.95 ± 8.18 years), and the incision length was 28.93 ± 18.02 mm. The control group (*n* = 40; 15 males, 25 females) underwent a conventional suture technique, with an age range of 20–51 years (mean ± standard deviation: 31.40 ± 6.63 years), and the incision length was 35.26 ± 20.19 mm. There were no statistically significant differences between the two groups in terms of age (*P* = 0.13), gender (*P* = 0.33), or incision length (*P* = 0.14) (*P* > 0.05).

Perioperative indicators: There was no statistically significant difference in the length of surgical incision between the two groups (*P* > 0.05). The operation time, duration of postoperative edema, and wound healing time of the experimental group were significantly shorter than those of the control group, which was statistically significant (*P* < 0.05). The treatment cost of the experimental group was significantly higher than that of the control group, which was statistically significant (*P* < 0.05) (Table 1).

**Table 1:** Comparison of perioperative parameters between the two groups.

Group	Incision length (mm)	Operation time (min)	Durability of edema (d)	Wound healing time (d)	Treatment cost (RMB)
Experimental group (*n* = 40)	28.93 ± 18.02	118.35 ± 33.03	5.74 ± 1.12	5.17 ± 0.48	2,215.03 ± 1,174.51
Control group (*n* = 40)	35.26 ± 20.19	45.35 ± 19.28	6.95 ± 1.18	6.89 ± 1.29	418.50 ± 170.95
*P*-value	0.14	<0.001	<0.001	<0.001	<0.001

Surgical complications: No complications were reported in the experimental group (reported incidence rate: 0%), while the complication rate in the control group was 18%. The chi-square test showed that the complication rate in the experimental group was significantly lower (*P* < 0.05) (Table 2).

**Table 2:** Comparison of complications between the two groups [*n* (%)].

Group	Hematoma	Infection	Knot reaction	Wound dehiscence	Dead space formation	Overall incidence (%)
Experimental group (*n* = 40)	0	0	0	0	0	0 (0%)
Control group (*n* = 40)	1	2	1	2	1	7 (18%)

Retrospective designs may result in minor complications going unrecorded.

Total satisfaction rate of treatment: After a systematic evaluation of 80 patients using a standard scale, it was found that the total satisfaction rate of the experimental group was 100%, of which 30 patients clearly stated that they were very satisfied with the treatment results, 10 patients agreed that the treatment results were relatively satisfactory, and no patient in the experimental group expressed dissatisfaction. The total satisfaction rate of the control group was 80%, and the chi-square test showed that the incidence of complications in the experimental group was significantly reduced (*P* < 0.05) (Table 3).

**Table 3:** Comparison of the total treatment satisfaction rate between the two groups [*n* (%)].

Group	Very satisfied	Satisfied	Dissatisfied	Overall satisfaction rate
Experimental group (*n* = 40)	30 (75.00)	10 (25.00)	0 (0.00)	40 (100.00)
Control group (*n* = 40)	12 (30.00)	20 (50.00)	8 (20.00)	32 (80.00)

Scar width: At 1, 3, 6 and 12 months after surgery, the scar width in the experimental group was significantly smaller than that in the control group (*P* < 0.05) (Table 4).

**Table 4:** Scar width.

Group	One month after surgery (mm)	Three months after surgery (mm)	Six months after surgery (mm)	12 months after surgery (mm)
Experimental group (*n* = 40)	0.19 ± 0.05	0.25 ± 0.04	0.31 ± 0.05	0.35 ± 0.05
Control group (*n* = 40)	2.84 ± 1.15	4.19 ± 1.64	4.81 ± 1.35	5.95 ± 1.76
*P*-value	<0.001	<0.001	<0.001	<0.001

All scar widths are measured in millimeters (mm).

Vancouver Scar Scale (VSS): At 1, 3, 6 and 12 months after surgery, the VSS scores of the experimental group were significantly lower than those of the control group (*P* < 0.05) (Table 5).

**Table 5:** Vancouver scar scale.

Group	One month after surgery	Three months after surgery	Six months after surgery	12 months after surgery
Experimental group (*n* = 40)	1.52 ± 0.68	3.02 ± 0.80	2.60 ± 0.59	2.02 ± 0.53
Control group (*n* = 40)	2.83 ± 1.24	4.05 ± 1.63	5.88 ± 1.59	4.83 ± 1.30
*P*-value	<0.001	<0.001	<0.001	<0.001

## Discussion

The superiority of cosmetic suturing is first reflected in its implementation of the concept of “tension-reducing suturing”. The principle of layered and fine tension-reducing suturing is an important feature that distinguishes cosmetic suturing from traditional suturing. In traditional suturing, the treatment of deep tissues is often ignored, resulting in the failure to effectively disperse subcutaneous tension, thereby affecting epidermal healing; while cosmetic suturing emphasizes the precise alignment of subcutaneous, dermal, and epidermal layers to make the wound smoother, promote the natural healing process of tissues, and reduce the risk of scar hyperplasia (21). Our study demonstrates its efficacy, with the experimental group showing significantly narrower scar widths (0.19 ± 0.05 mm vs. 2.84 ± 1.15 mm at 1 month, *P* < 0.001, Table 4). These results align with prior findings that highlight the importance of tension control in optimizing aesthetic outcomes. In addition, this procedure pays special attention to following the direction of skin texture (such as Langer's line) when suturing, and adjusts the suturing strategy according to the movement characteristics of specific parts such as eyelids and mouth, making the wound less likely to split or pull during dynamic facial activities, thereby further optimizing the postoperative appearance (22).

Tension is one of the key factors in scar formation (23). Traditional suturing often results in poor wound alignment or even skin edge necrosis due to rough operation or improper control of suture tension by the surgeon, ultimately forming obvious scars. During the suturing process, cosmetic suturing reduces pulling, avoids clamping, and uses fine sutures (5-0/6-0 absorbable for muscle/subcutaneous layers, 7-0 nylon/Prolene for epidermis) and precise needle spacing (0.5–1 cm) to achieve minimal trauma and invisible scar suturing effects.Relevant clinical studies have pointed out that the use of fine instruments combined with professional suturing techniques can help minimize intraoperative trauma and improve postoperative healing quality (24).This finding is consistent with the conclusions of our study.

In this study, the Vancouver Scar Scale (VSS) provided a robust framework to quantify scar quality, assessing color, vascularity, pliability, and height (0–15 scale) (20). Our results showed that the experimental group had significantly lower VSS scores across all time points (1.52 ± 0.68, 3.02 ± 0.80, 2.60 ± 0.59, 2.02 ± 0.53 at 1, 3, 6, and 12 months) compared to the control group (2.83 ± 1.24, 4.05 ± 1.63, 5.88 ± 1.59, 4.83 ± 1.30, *P* < 0.001, Table 5). This confirms the technique's efficacy in inhibiting scar hyperplasia and improving texture, consistent with prior studies (25). The VSS's standardized metrics offer clinicians a reliable tool to tailor surgical strategies, enhancing patient outcomes. However, these clinical benefits must be weighed against the increased treatment costs.

The significantly higher treatment costs in the experimental group (2,215.03 ± 1,174.51 RMB vs. 418.50 ± 170.95 RMB, *P* < 0.001, Table 1) warrant detailed examination. Key drivers include: (1) hospital pricing policy leading to higher costs for larger wounds: According to the hospital's fee schedule, the base fee for incisions up to 3 cm is approximately 1,500 RMB, with an additional charge of 500–1,000 RMB for each subsequent 1 cm of incision length; (2) premium suture materials, such as 5-0/6-0 absorbable sutures and 7-0 nylon/Prolene, which increase material costs by approximately 20%–30% compared to traditional sutures; and (3) extended operative times (118.35 ± 33.03 min vs. 45.35 ± 19.28 min, *P* < 0.001), particularly for larger wounds requiring meticulous layered suturing and adjunct techniques (e.g., V-Y advancement, 3M tape). Despite higher upfront costs, the experimental group's low complication rate and higher satisfaction levels indicate potential long-term cost savings through reduced revision surgeries or scar treatments. This conclusion aligns with findings from related studies (5, 7).

Although this study primarily focuses on fresh, regular facial soft tissue injuries—which typically yield high cosmetic suturing success rates—the practical applicability of tension-reducing suturing techniques extends to more complex wound types, such as irregular defects or extensive tissue defects. When managing these complex cases, the technique can serve as a core suturing strategy. By employing a layered approach—progressively reducing tension from the muscular layer to the epidermis—it evenly distributes stress, thereby minimizing risks of postoperative hypertrophic scarring, wound dehiscence, and deformity (7). Specifically, for irregular defects, V-Y advancement or local rotation flaps can first repair the deficient area, followed by tension-reducing suturing to ensure tension-free edge apposition. For extensive tissue defects, free skin grafts or biomaterial fillers may be combined with subcutaneous tension reduction using 5-0 or 6-0 absorbable sutures to promote natural healing and minimize dead space formation (18). However, applying this technique to complex wounds may present several challenges, including uneven tension distribution (particularly at irregular margins), the need for additional reconstruction of deep tissue damage (e.g., fascial repair or vascular anastomosis), and increased risk of postoperative complications (e.g., infection or hematoma, especially in cases of high contamination). Literature evidence indicates that tension-relieving suturing excels in facial scar management. For instance, in Asian patients, continuous tension-relieving techniques significantly improved aesthetic outcomes and reduced scar width (7). In major wound closure, super-tension-relieving suturing combined with slow-absorbing materials further enhanced patient prognosis (26). Nevertheless, this study's limitations include strict exclusion criteria for complex cases (e.g., extensive trauma or open fractures), rendering results primarily applicable to simple wounds. Future multicenter, prospective studies should incorporate diverse wound types to systematically evaluate this technique's efficacy, safety, and clinical applicability in irregular or large defects, thereby providing evidence-based support for broader maxillofacial reconstructive practice.

In the future, efforts should focus on promoting high-quality multicenter clinical research, expanding sample coverage, extending observation periods, and systematically evaluating the long-term efficacy and safety characteristics of interventions across three dimensions: patients with different injury mechanisms, varying injury severity, and different injury locations. However, this study has certain limitations. First, the relatively small sample size and single-center origin may limit the generalizability of the findings. Second, the retrospective design and reliance on clinical observation may have led to underreporting of minor complications (e.g., mild inflammation or microhematomas), as imaging or biomarkers were not routinely employed for detection. Validating the reported 0% complication rate in the experimental group requires larger sample sizes and more sensitive diagnostic methods. Additionally, the follow-up period was insufficient to comprehensively evaluate long-term scar remodeling, and potential biases related to patient compliance and subjective scar appearance assessments were difficult to fully mitigate. Furthermore, treatment cost data may exhibit regional variations influenced by hospital pricing and material costs, and indirect costs (such as patient recovery time) were not evaluated. Finally, while tension-relieving suturing techniques offer significant advantages in maxillofacial reconstructive surgery (particularly for simple wounds), their application to complex wounds requires further exploration as part of a comprehensive strategy, with additional studies needed to validate their efficacy. These limitations underscore the necessity for larger-scale, prospective, and long-term studies to validate and refine the conclusions drawn from this research.

## Conclusion

The application of tension-relieving suture technology in maxillofacial plastic surgery has shown significant advantages. It can not only effectively reduce the incidence of surgical complications, but also significantly reduce scar width. In summary, this technology has important clinical value in the field of maxillofacial plastic surgery and is worthy of promotion and application.

## Data Availability

The original contributions presented in the study are included in the article/Supplementary Material, further inquiries can be directed to the corresponding authors.
