# Supplementary material for: Investigating the efficacy of the application of tension-reducing suture technique in maxillofacial plastic and cosmetic surgery
Source: Front Surg. 2025 Oct 10;12:1669865. doi: 10.3389/fsurg.2025.1669865 (PMC12549582; doi:10.3389/fsurg.2025.1669865)
Supplement: Supplementary file 1 [file Supplementaryfile1.docx]

[**Supplementary materials**](https://review.frontiersin.org/Document/DownloadSupplementaryMaterial?articleId=1669865&userId=1049555&roleId=16#tab/_blank)**: Raw data**

**Comparison of Perioperative Parameters Between Two Groups (Including Gender and Age)**

| **Experimental group (n=40) raw data** |  |  |  |  |  |  |  |
| --- | --- | --- | --- | --- | --- | --- | --- |
| **Patient ID** | **Incision length (mm)** | **Operation time (min)** | **Durability of edema (d)** | **Wound healing time (d)** | **Treatment cost (RMB)** | **Age (Years)** | **Gender** |
| **1** | **41.9** | **163** | **6.8** | **6** | **2530** | **19** | **Female** |
| **2** | **27.3** | **124** | **5.9** | **5.1** | **1715** | **34** | **Female** |
| **3** | **45.4** | **150** | **8.5** | **5.5** | **2798** | **39** | **Female** |
| **4** | **65.6** | **163** | **5.4** | **6.1** | **5024** | **42** | **Female** |
| **5** | **25.2** | **127** | **6.1** | **4.4** | **1585** | **24** | **Female** |
| **6** | **20.5** | **100** | **5.4** | **4** | **1551** | **35** | **Female** |
| **7** | **66.9** | **181** | **6.7** | **5.6** | **4987** | **43** | **Female** |
| **8** | **48.2** | **167** | **8** | **4.5** | **3397** | **35** | **Female** |
| **9** | **19.7** | **94** | **5.4** | **7.3** | **1618** | **33** | **Female** |
| **10** | **43** | **151** | **4.5** | **4.8** | **2835** | **23** | **Female** |
| **11** | **19.8** | **111** | **6.3** | **6.2** | **1522** | **53** | **Female** |
| **12** | **19.3** | **107** | **4.6** | **4.5** | **1594** | **30** | **Female** |
| **13** | **36.1** | **125** | **5.5** | **5.8** | **2108** | **40** | **Female** |
| **14** | **12.7** | **77** | **6.2** | **5.9** | **1500** | **36** | **Female** |
| **15** | **12.6** | **82** | **5.3** | **4.7** | **1675** | **36** | **Male** |
| **16** | **17.6** | **76** | **6** | **6.9** | **1565** | **23** | **Female** |
| **17** | **7.4** | **93** | **5.6** | **4.8** | **1555** | **36** | **Male** |
| **18** | **37.8** | **137** | **5.3** | **4.2** | **2249** | **30** | **Male** |
| **19** | **9.6** | **86** | **5.3** | **4.8** | **1590** | **35** | **Male** |
| **20** | **14** | **96** | **7.7** | **7** | **1670** | **37** | **Female** |
| **21** | **72.3** | **188** | **8.1** | **6.3** | **5622** | **24** | **Male** |
| **22** | **25.3** | **112** | **5.6** | **5** | **1706** | **28** | **Male** |
| **23** | **32.1** | **122** | **5** | **4.5** | **1773** | **31** | **Male** |
| **24** | **13.7** | **74** | **5.7** | **5** | **1510** | **35** | **Male** |
| **25** | **18** | **87** | **4.8** | **5.2** | **1537** | **31** | **Male** |
| **26** | **33.1** | **134** | **6.9** | **5.2** | **1784** | **27** | **Female** |
| **27** | **7** | **99** | **5.3** | **4.6** | **1396** | **32** | **Female** |
| **28** | **39.2** | **136** | **4.6** | **4.5** | **2224** | **44** | **Female** |
| **29** | **16.7** | **106** | **6.2** | **4.6** | **1500** | **33** | **Male** |
| **30** | **23.8** | **105** | **6.5** | **5.5** | **1545** | **37** | **Female** |
| **31** | **16.6** | **86** | **4.4** | **4.6** | **1606** | **21** | **Female** |
| **32** | **72.2** | **201** | **7.6** | **6.3** | **5851** | **31** | **Female** |
| **33** | **30.2** | **112** | **4.9** | **4.8** | **1753** | **33** | **Female** |
| **34** | **6.2** | **94** | **4.6** | **4.4** | **1547** | **51** | **Female** |
| **35** | **47.7** | **145** | **6.2** | **5.6** | **3273** | **36** | **Female** |
| **36** | **14.2** | **80** | **4.9** | **4.5** | **1662** | **34** | **Female** |
| **37** | **35.3** | **138** | **4.4** | **4.6** | **2056** | **54** | **Female** |
| **38** | **12.8** | **89** | **4.6** | **4.2** | **1560** | **41** | **Female** |
| **39** | **15.1** | **97** | **4.4** | **4.3** | **1664** | **31** | **Female** |
| **40** | **35.1** | **127** | **4.2** | **4.9** | **1964** | **21** | **Female** |

| **Control group (n=40) raw data** |  |  |  |  |  |  |  |
| --- | --- | --- | --- | --- | --- | --- | --- |
| **Patient ID** | **Incision length (mm)** | **Operation time (min)** | **Durability of edema (d)** | **Wound healing time (d)** | **Treatment cost (RMB)** | **Age (Years)** | **Gender** |
| **1** | **39.3** | **33** | **6.6** | **6.9** | **399** | **33** | **Female** |
| **2** | **40.3** | **56** | **7.6** | **7.8** | **459** | **33** | **Female** |
| **3** | **51.7** | **66** | **9.2** | **6.2** | **461** | **33** | **Female** |
| **4** | **50.9** | **65** | **7.8** | **9.2** | **492** | **36** | **Female** |
| **5** | **2.5** | **23** | **3.2** | **6.1** | **309** | **20** | **Male** |
| **6** | **11.9** | **34** | **3** | **4.4** | **274** | **27** | **Male** |
| **7** | **40.2** | **56** | **6.3** | **8.2** | **281** | **34** | **Female** |
| **8** | **36.9** | **37** | **7.4** | **8** | **269** | **32** | **Female** |
| **9** | **40.6** | **53** | **7** | **7.7** | **274** | **33** | **Female** |
| **10** | **101.9** | **98** | **12.8** | **9.6** | **1273** | **51** | **Female** |
| **11** | **42.7** | **49** | **6.2** | **6.7** | **369** | **33** | **Female** |
| **12** | **52.2** | **67** | **5.9** | **5.4** | **598** | **39** | **Female** |
| **13** | **48.2** | **47** | **5.7** | **6.7** | **454** | **35** | **Female** |
| **14** | **44.7** | **60** | **7.9** | **6.2** | **424** | **35** | **Male** |
| **15** | **26.9** | **36** | **6.8** | **8.2** | **350** | **28** | **Male** |
| **16** | **47.3** | **59** | **5.5** | **6.4** | **423** | **33** | **Male** |
| **17** | **14.8** | **24** | **7.4** | **6** | **292** | **26** | **Male** |
| **18** | **26.1** | **37** | **6.7** | **6.4** | **359** | **28** | **Female** |
| **19** | **22.4** | **35** | **7.4** | **7.6** | **368** | **27** | **Male** |
| **20** | **34.4** | **34** | **7.3** | **6.2** | **320** | **30** | **Female** |
| **21** | **74.7** | **84** | **10.6** | **7.4** | **618** | **47** | **Female** |
| **22** | **4.7** | **24** | **4.8** | **6.9** | **287** | **21** | **Male** |
| **23** | **42.3** | **50** | **7.7** | **7.4** | **455** | **33** | **Female** |
| **24** | **2.9** | **18** | **6.7** | **6.3** | **285** | **22** | **Male** |
| **25** | **23.5** | **28** | **6.1** | **6.1** | **323** | **27** | **Male** |
| **26** | **54.4** | **77** | **6.9** | **7.1** | **577** | **36** | **Female** |
| **27** | **32.3** | **31** | **8.3** | **5.2** | **489** | **32** | **Female** |
| **28** | **12.3** | **18** | **5** | **4.7** | **359** | **26** | **Male** |
| **29** | **18** | **25** | **7** | **6** | **324** | **26** | **Male** |
| **30** | **43.1** | **51** | **6.2** | **6.4** | **434** | **34** | **Female** |
| **31** | **17.7** | **26** | **5.7** | **7.1** | **295** | **26** | **Male** |
| **32** | **38** | **42** | **8.1** | **9.2** | **497** | **28** | **Female** |
| **33** | **31.9** | **41** | **7.6** | **8.2** | **390** | **28** | **Male** |
| **34** | **21.4** | **30** | **7.4** | **6.9** | **377** | **29** | **Male** |
| **35** | **67.7** | **80** | **11.1** | **10.2** | **638** | **45** | **Female** |
| **36** | **44.8** | **56** | **6.6** | **5.4** | **440** | **34** | **Female** |
| **37** | **8.1** | **29** | **5.7** | **5.9** | **253** | **20** | **Female** |
| **38** | **34.2** | **40** | **5.9** | **6.3** | **449** | **32** | **Female** |
| **39** | **18.6** | **34** | **6.6** | **7.3** | **361** | **27** | **Female** |
| **40** | **44** | **61** | **6.4** | **5.7** | **441** | **37** | **Female** |

**Scar width**

| **Experimental group (n=40) Scar width raw data (unit: mm)** |  |  |  |  |
| --- | --- | --- | --- | --- |
| **Patient ID** | **One month after surgery** | **Three months after surgery** | **Six months after surgery** | **12 months after surgery** |
| **1** | **0.23** | **0.24** | **0.25** | **0.31** |
| **2** | **0.19** | **0.27** | **0.34** | **0.35** |
| **3** | **0.23** | **0.33** | **0.48** | **0.38** |
| **4** | **0.28** | **0.27** | **0.26** | **0.39** |
| **5** | **0.18** | **0.21** | **0.34** | **0.3** |
| **6** | **0.19** | **0.23** | **0.32** | **0.33** |
| **7** | **0.26** | **0.29** | **0.34** | **0.33** |
| **8** | **0.24** | **0.27** | **0.4** | **0.32** |
| **9** | **0.18** | **0.22** | **0.29** | **0.44** |
| **10** | **0.22** | **0.27** | **0.27** | **0.37** |
| **11** | **0.18** | **0.25** | **0.26** | **0.29** |
| **12** | **0.18** | **0.3** | **0.26** | **0.39** |
| **13** | **0.21** | **0.21** | **0.3** | **0.45** |
| **14** | **0.11** | **0.23** | **0.32** | **0.4** |
| **15** | **0.12** | **0.23** | **0.31** | **0.27** |
| **16** | **0.17** | **0.18** | **0.34** | **0.32** |
| **17** | **0.15** | **0.26** | **0.3** | **0.42** |
| **18** | **0.21** | **0.26** | **0.37** | **0.32** |
| **19** | **0.16** | **0.25** | **0.29** | **0.37** |
| **20** | **0.13** | **0.24** | **0.44** | **0.39** |
| **21** | **0.27** | **0.18** | **0.39** | **0.39** |
| **22** | **0.19** | **0.23** | **0.25** | **0.35** |
| **23** | **0.2** | **0.23** | **0.25** | **0.19** |
| **24** | **0.13** | **0.21** | **0.32** | **0.3** |
| **25** | **0.17** | **0.24** | **0.29** | **0.34** |
| **26** | **0.21** | **0.27** | **0.34** | **0.29** |
| **27** | **0.14** | **0.34** | **0.32** | **0.43** |
| **28** | **0.22** | **0.26** | **0.29** | **0.28** |
| **29** | **0.17** | **0.26** | **0.26** | **0.33** |
| **30** | **0.18** | **0.25** | **0.23** | **0.36** |
| **31** | **0.17** | **0.15** | **0.28** | **0.42** |
| **32** | **0.3** | **0.25** | **0.34** | **0.28** |
| **33** | **0.2** | **0.25** | **0.31** | **0.41** |
| **34** | **0.15** | **0.37** | **0.24** | **0.35** |
| **35** | **0.23** | **0.24** | **0.31** | **0.3** |
| **36** | **0.14** | **0.26** | **0.32** | **0.37** |
| **37** | **0.21** | **0.25** | **0.25** | **0.36** |
| **38** | **0.1** | **0.19** | **0.31** | **0.32** |
| **39** | **0.13** | **0.3** | **0.3** | **0.35** |
| **40** | **0.2** | **0.29** | **0.24** | **0.33** |
|  |  |  |  |  |

| **Control group (n=40) Scar width raw data (unit: mm)** |  |  |  |  |
| --- | --- | --- | --- | --- |
| **Patient ID** | **One month after surgery** | **Three months after surgery** | **Six months after surgery** | **12 months after surgery** |
| **1** | **3.18** | **1.64** | **4.68** | **6.49** |
| **2** | **4.01** | **3.1** | **3.61** | **9.04** |
| **3** | **1.76** | **3.85** | **2.54** | **7.37** |
| **4** | **6.17** | **4.06** | **5.35** | **5.61** |
| **5** | **1.54** | **2.23** | **3.81** | **2.83** |
| **6** | **1.15** | **4.98** | **2.73** | **5.53** |
| **7** | **4.75** | **2.43** | **3.91** | **2.88** |
| **8** | **4.19** | **3.72** | **3.38** | **4.39** |
| **9** | **3.94** | **4.14** | **7.55** | **3.06** |
| **10** | **3.88** | **6.20** | **6.25** | **5.58** |
| **11** | **2.98** | **5.06** | **5.04** | **6.08** |
| **12** | **1.69** | **2.38** | **7.1** | **8.51** |
| **13** | **3.22** | **1.62** | **5.11** | **6.51** |
| **14** | **2.06** | **5.95** | **3.62** | **5.59** |
| **15** | **4.62** | **4.46** | **7.24** | **7.43** |
| **16** | **2.72** | **2.82** | **5.81** | **2.69** |
| **17** | **1.83** | **6.32** | **3.38** | **6.61** |
| **18** | **2.53** | **4.1** | **4.69** | **7.21** |
| **19** | **3.78** | **5.73** | **3.76** | **3.82** |
| **20** | **2.09** | **4.01** | **4.69** | **7.73** |
| **21** | **1.7** | **7.24** | **6.45** | **6.45** |
| **22** | **3.32** | **6.64** | **7.78** | **5.41** |
| **23** | **3.21** | **3.57** | **2.94** | **7.09** |
| **24** | **2.2** | **5.47** | **5.95** | **11.20** |
| **25** | **2.24** | **4.96** | **3.84** | **6.39** |
| **26** | **3.36** | **6.04** | **4.30** | **6.33** |
| **27** | **1.03** | **2.6** | **4.15** | **3.24** |
| **28** | **1.14** | **5.09** | **3.69** | **4.72** |
| **29** | **1.99** | **5.55** | **5.1** | **7.25** |
| **30** | **2.64** | **1.32** | **3.71** | **4.8** |
| **31** | **1.86** | **2.2** | **5.41** | **5.98** |
| **32** | **4.25** | **4.83** | **5.65** | **6.4** |
| **33** | **4.19** | **3.48** | **4.73** | **6.71** |
| **34** | **2.9** | **5.18** | **3.66** | **6.47** |
| **35** | **3.06** | **6.38** | **4.16** | **7.48** |
| **36** | **1.46** | **4.09** | **6.1** | **5.11** |
| **37** | **2.84** | **6.49** | **5.71** | **5.66** |
| **38** | **2.67** | **1.95** | **3.5** | **4.54** |
| **39** | **3.48** | **1.68** | **5.18** | **5.24** |
| **40** | **1.85** | **4** | **6.1** | **6.47** |

**VSS Score**

| **Patient ID** | **Group** | **Time Point** | **Total VSS** | **Pigmentation (0-3)** | **Vascularity (0-3)** | **Height (0-4)** | **Pliability (0-5)** |
| --- | --- | --- | --- | --- | --- | --- | --- |
| **1** | **Experimental** | **1 Month** | **2** | **0** | **1** | **0** | **1** |
| **1** | **Experimental** | **3 Months** | **2** | **0** | **1** | **0** | **1** |
| **1** | **Experimental** | **6 Months** | **2** | **0** | **1** | **0** | **1** |
| **1** | **Experimental** | **12 Months** | **2** | **0** | **0** | **1** | **1** |
| **1** | **Control** | **1 Month** | **3** | **0** | **2** | **0** | **1** |
| **1** | **Control** | **3 Months** | **2** | **0** | **1** | **0** | **1** |
| **1** | **Control** | **6 Months** | **6** | **1** | **1** | **2** | **2** |
| **1** | **Control** | **12 Months** | **5** | **1** | **1** | **1** | **2** |
| **2** | **Experimental** | **1 Month** | **1** | **0** | **0** | **0** | **1** |
| **2** | **Experimental** | **3 Months** | **3** | **1** | **1** | **1** | **0** |
| **2** | **Experimental** | **6 Months** | **3** | **1** | **1** | **0** | **1** |
| **2** | **Experimental** | **12 Months** | **1** | **0** | **0** | **0** | **1** |
| **2** | **Control** | **1 Month** | **4** | **1** | **1** | **1** | **1** |
| **2** | **Control** | **3 Months** | **3** | **0** | **1** | **1** | **1** |
| **2** | **Control** | **6 Months** | **9** | **1** | **2** | **3** | **3** |
| **2** | **Control** | **12 Months** | **4** | **1** | **0** | **1** | **2** |
| **3** | **Experimental** | **1 Month** | **2** | **0** | **1** | **1** | **0** |
| **3** | **Experimental** | **3 Months** | **3** | **1** | **1** | **0** | **1** |
| **3** | **Experimental** | **6 Months** | **3** | **1** | **1** | **0** | **1** |
| **3** | **Experimental** | **12 Months** | **3** | **1** | **1** | **0** | **1** |
| **3** | **Control** | **1 Month** | **2** | **0** | **1** | **0** | **1** |
| **3** | **Control** | **3 Months** | **4** | **1** | **1** | **1** | **1** |
| **3** | **Control** | **6 Months** | **7** | **1** | **1** | **2** | **3** |
| **3** | **Control** | **12 Months** | **3** | **0** | **0** | **1** | **2** |
| **4** | **Experimental** | **1 Month** | **3** | **1** | **1** | **0** | **1** |
| **4** | **Experimental** | **3 Months** | **3** | **1** | **1** | **0** | **1** |
| **4** | **Experimental** | **6 Months** | **2** | **0** | **1** | **0** | **1** |
| **4** | **Experimental** | **12 Months** | **3** | **0** | **1** | **1** | **1** |
| **4** | **Control** | **1 Month** | **6** | **1** | **2** | **1** | **2** |
| **4** | **Control** | **3 Months** | **4** | **1** | **1** | **1** | **1** |
| **4** | **Control** | **6 Months** | **6** | **1** | **1** | **2** | **2** |
| **4** | **Control** | **12 Months** | **5** | **0** | **1** | **2** | **2** |
| **5** | **Experimental** | **1 Month** | **1** | **0** | **1** | **0** | **0** |
| **5** | **Experimental** | **3 Months** | **3** | **1** | **1** | **0** | **1** |
| **5** | **Experimental** | **6 Months** | **3** | **1** | **1** | **0** | **1** |
| **5** | **Experimental** | **12 Months** | **2** | **0** | **0** | **1** | **1** |
| **5** | **Control** | **1 Month** | **1** | **0** | **1** | **0** | **0** |
| **5** | **Control** | **3 Months** | **3** | **1** | **1** | **0** | **1** |
| **5** | **Control** | **6 Months** | **3** | **0** | **1** | **1** | **1** |
| **5** | **Control** | **12 Months** | **4** | **0** | **1** | **1** | **2** |
| **6** | **Experimental** | **1 Month** | **1** | **0** | **1** | **0** | **0** |
| **6** | **Experimental** | **3 Months** | **3** | **1** | **1** | **1** | **0** |
| **6** | **Experimental** | **6 Months** | **3** | **1** | **1** | **0** | **1** |
| **6** | **Experimental** | **12 Months** | **2** | **0** | **1** | **0** | **1** |
| **6** | **Control** | **1 Month** | **1** | **0** | **1** | **0** | **0** |
| **6** | **Control** | **3 Months** | **5** | **1** | **1** | **1** | **2** |
| **6** | **Control** | **6 Months** | **6** | **1** | **1** | **2** | **2** |
| **6** | **Control** | **12 Months** | **3** | **0** | **1** | **1** | **1** |
| **7** | **Experimental** | **1 Month** | **3** | **1** | **1** | **0** | **1** |
| **7** | **Experimental** | **3 Months** | **3** | **1** | **1** | **0** | **1** |
| **7** | **Experimental** | **6 Months** | **3** | **1** | **1** | **0** | **1** |
| **7** | **Experimental** | **12 Months** | **3** | **1** | **1** | **0** | **1** |
| **7** | **Control** | **1 Month** | **5** | **1** | **2** | **0** | **2** |
| **7** | **Control** | **3 Months** | **2** | **0** | **1** | **0** | **1** |
| **7** | **Control** | **6 Months** | **3** | **0** | **1** | **1** | **1** |
| **7** | **Control** | **12 Months** | **4** | **1** | **1** | **1** | **1** |
| **8** | **Experimental** | **1 Month** | **2** | **0** | **1** | **0** | **1** |
| **8** | **Experimental** | **3 Months** | **3** | **1** | **1** | **0** | **1** |
| **8** | **Experimental** | **6 Months** | **4** | **1** | **1** | **1** | **1** |
| **8** | **Experimental** | **12 Months** | **2** | **0** | **0** | **1** | **1** |
| **8** | **Control** | **1 Month** | **4** | **1** | **1** | **1** | **1** |
| **8** | **Control** | **3 Months** | **4** | **1** | **1** | **1** | **1** |
| **8** | **Control** | **6 Months** | **4** | **1** | **1** | **1** | **1** |
| **8** | **Control** | **12 Months** | **3** | **1** | **0** | **1** | **1** |
| **9** | **Experimental** | **1 Month** | **1** | **0** | **1** | **0** | **0** |
| **9** | **Experimental** | **3 Months** | **4** | **1** | **1** | **1** | **1** |
| **9** | **Experimental** | **6 Months** | **2** | **1** | **1** | **0** | **0** |
| **9** | **Experimental** | **12 Months** | **2** | **0** | **1** | **0** | **1** |
| **9** | **Control** | **1 Month** | **4** | **1** | **2** | **0** | **1** |
| **9** | **Control** | **3 Months** | **4** | **1** | **1** | **1** | **1** |
| **9** | **Control** | **6 Months** | **3** | **0** | **1** | **1** | **1** |
| **9** | **Control** | **12 Months** | **8** | **1** | **1** | **3** | **3** |
| **10** | **Experimental** | **1 Month** | **2** | **0** | **1** | **1** | **0** |
| **10** | **Experimental** | **3 Months** | **3** | **1** | **1** | **1** | **0** |
| **10** | **Experimental** | **6 Months** | **2** | **1** | **1** | **0** | **0** |
| **10** | **Experimental** | **12 Months** | **2** | **0** | **1** | **0** | **1** |
| **10** | **Control** | **1 Month** | **4** | **1** | **1** | **1** | **1** |
| **10** | **Control** | **3 Months** | **5** | **1** | **1** | **1** | **2** |
| **10** | **Control** | **6 Months** | **6** | **1** | **1** | **2** | **2** |
| **10** | **Control** | **12 Months** | **6** | **1** | **1** | **2** | **2** |
| **11** | **Experimental** | **1 Month** | **1** | **0** | **1** | **0** | **0** |
| **11** | **Experimental** | **3 Months** | **2** | **0** | **1** | **0** | **1** |
| **11** | **Experimental** | **6 Months** | **2** | **1** | **1** | **0** | **0** |
| **11** | **Experimental** | **12 Months** | **2** | **0** | **1** | **0** | **1** |
| **11** | **Control** | **1 Month** | **3** | **1** | **1** | **0** | **1** |
| **11** | **Control** | **3 Months** | **5** | **1** | **1** | **1** | **2** |
| **11** | **Control** | **6 Months** | **6** | **1** | **1** | **2** | **2** |
| **11** | **Control** | **12 Months** | **5** | **1** | **1** | **1** | **2** |
| **12** | **Experimental** | **1 Month** | **1** | **0** | **0** | **1** | **0** |
| **12** | **Experimental** | **3 Months** | **3** | **1** | **1** | **1** | **0** |
| **12** | **Experimental** | **6 Months** | **2** | **0** | **1** | **0** | **1** |
| **12** | **Experimental** | **12 Months** | **3** | **1** | **1** | **0** | **1** |
| **12** | **Control** | **1 Month** | **2** | **0** | **1** | **0** | **1** |
| **12** | **Control** | **3 Months** | **2** | **0** | **1** | **0** | **1** |
| **12** | **Control** | **6 Months** | **9** | **1** | **2** | **3** | **3** |
| **12** | **Control** | **12 Months** | **7** | **1** | **1** | **2** | **3** |
| **13** | **Experimental** | **1 Month** | **2** | **0** | **1** | **0** | **1** |
| **13** | **Experimental** | **3 Months** | **6** | **1** | **2** | **1** | **2** |
| **13** | **Experimental** | **6 Months** | **3** | **1** | **1** | **0** | **1** |
| **13** | **Experimental** | **12 Months** | **2** | **0** | **1** | **0** | **1** |
| **13** | **Control** | **1 Month** | **3** | **1** | **1** | **0** | **1** |
| **13** | **Control** | **3 Months** | **2** | **0** | **1** | **0** | **1** |
| **13** | **Control** | **6 Months** | **7** | **1** | **1** | **2** | **3** |
| **13** | **Control** | **12 Months** | **5** | **0** | **1** | **2** | **2** |
| **14** | **Experimental** | **1 Month** | **1** | **0** | **0** | **1** | **0** |
| **14** | **Experimental** | **3 Months** | **4** | **1** | **1** | **1** | **1** |
| **14** | **Experimental** | **6 Months** | **3** | **1** | **1** | **0** | **1** |
| **14** | **Experimental** | **12 Months** | **2** | **0** | **1** | **0** | **1** |
| **14** | **Control** | **1 Month** | **2** | **0** | **1** | **0** | **1** |
| **14** | **Control** | **3 Months** | **6** | **1** | **2** | **1** | **2** |
| **14** | **Control** | **6 Months** | **6** | **1** | **1** | **2** | **2** |
| **14** | **Control** | **12 Months** | **4** | **0** | **1** | **1** | **2** |
| **15** | **Experimental** | **1 Month** | **1** | **0** | **1** | **0** | **0** |
| **15** | **Experimental** | **3 Months** | **2** | **0** | **1** | **0** | **1** |
| **15** | **Experimental** | **6 Months** | **3** | **1** | **1** | **0** | **1** |
| **15** | **Experimental** | **12 Months** | **2** | **0** | **1** | **0** | **1** |
| **15** | **Control** | **1 Month** | **5** | **1** | **1** | **1** | **2** |
| **15** | **Control** | **3 Months** | **4** | **1** | **1** | **1** | **1** |
| **15** | **Control** | **6 Months** | **7** | **1** | **1** | **2** | **3** |
| **15** | **Control** | **12 Months** | **7** | **1** | **1** | **2** | **3** |
| **16** | **Experimental** | **1 Month** | **1** | **0** | **0** | **1** | **0** |
| **16** | **Experimental** | **3 Months** | **3** | **1** | **1** | **0** | **1** |
| **16** | **Experimental** | **6 Months** | **3** | **1** | **1** | **0** | **1** |
| **16** | **Experimental** | **12 Months** | **1** | **0** | **0** | **0** | **1** |
| **16** | **Control** | **1 Month** | **3** | **1** | **1** | **0** | **1** |
| **16** | **Control** | **3 Months** | **3** | **1** | **1** | **0** | **1** |
| **16** | **Control** | **6 Months** | **3** | **0** | **1** | **1** | **1** |
| **16** | **Control** | **12 Months** | **6** | **1** | **1** | **2** | **2** |
| **17** | **Experimental** | **1 Month** | **1** | **0** | **1** | **0** | **0** |
| **17** | **Experimental** | **3 Months** | **4** | **1** | **1** | **1** | **1** |
| **17** | **Experimental** | **6 Months** | **3** | **1** | **1** | **0** | **1** |
| **17** | **Experimental** | **12 Months** | **2** | **0** | **1** | **0** | **1** |
| **17** | **Control** | **1 Month** | **2** | **0** | **1** | **0** | **1** |
| **17** | **Control** | **3 Months** | **6** | **1** | **2** | **1** | **2** |
| **17** | **Control** | **6 Months** | **5** | **1** | **1** | **1** | **2** |
| **17** | **Control** | **12 Months** | **3** | **0** | **1** | **1** | **1** |
| **18** | **Experimental** | **1 Month** | **2** | **0** | **1** | **0** | **1** |
| **18** | **Experimental** | **3 Months** | **3** | **1** | **1** | **0** | **1** |
| **18** | **Experimental** | **6 Months** | **3** | **1** | **1** | **0** | **1** |
| **18** | **Experimental** | **12 Months** | **2** | **0** | **1** | **0** | **1** |
| **18** | **Control** | **1 Month** | **3** | **1** | **1** | **0** | **1** |
| **18** | **Control** | **3 Months** | **4** | **1** | **1** | **1** | **1** |
| **18** | **Control** | **6 Months** | **7** | **1** | **1** | **2** | **3** |
| **18** | **Control** | **12 Months** | **5** | **1** | **1** | **1** | **2** |
| **19** | **Experimental** | **1 Month** | **1** | **0** | **0** | **1** | **0** |
| **19** | **Experimental** | **3 Months** | **3** | **1** | **1** | **0** | **1** |
| **19** | **Experimental** | **6 Months** | **2** | **0** | **1** | **0** | **1** |
| **19** | **Experimental** | **12 Months** | **2** | **0** | **1** | **0** | **1** |
| **19** | **Control** | **1 Month** | **4** | **1** | **1** | **1** | **1** |
| **19** | **Control** | **3 Months** | **6** | **1** | **2** | **1** | **2** |
| **19** | **Control** | **6 Months** | **4** | **0** | **1** | **1** | **2** |
| **19** | **Control** | **12 Months** | **4** | **1** | **1** | **1** | **1** |
| **20** | **Experimental** | **1 Month** | **1** | **0** | **1** | **0** | **0** |
| **20** | **Experimental** | **3 Months** | **3** | **1** | **1** | **0** | **1** |
| **20** | **Experimental** | **6 Months** | **4** | **1** | **1** | **1** | **1** |
| **20** | **Experimental** | **12 Months** | **2** | **0** | **0** | **1** | **1** |
| **20** | **Control** | **1 Month** | **2** | **0** | **1** | **0** | **1** |
| **20** | **Control** | **3 Months** | **4** | **1** | **1** | **1** | **1** |
| **20** | **Control** | **6 Months** | **8** | **1** | **1** | **3** | **3** |
| **20** | **Control** | **12 Months** | **5** | **1** | **1** | **1** | **2** |
| **21** | **Experimental** | **1 Month** | **3** | **1** | **1** | **0** | **1** |
| **21** | **Experimental** | **3 Months** | **3** | **1** | **1** | **1** | **0** |
| **21** | **Experimental** | **6 Months** | **2** | **1** | **1** | **0** | **0** |
| **21** | **Experimental** | **12 Months** | **1** | **0** | **0** | **0** | **1** |
| **21** | **Control** | **1 Month** | **2** | **0** | **1** | **0** | **1** |
| **21** | **Control** | **3 Months** | **7** | **1** | **2** | **1** | **3** |
| **21** | **Control** | **6 Months** | **6** | **1** | **1** | **2** | **2** |
| **21** | **Control** | **12 Months** | **6** | **1** | **1** | **2** | **2** |
| **22** | **Experimental** | **1 Month** | **1** | **0** | **1** | **0** | **0** |
| **22** | **Experimental** | **3 Months** | **3** | **1** | **1** | **0** | **1** |
| **22** | **Experimental** | **6 Months** | **2** | **1** | **1** | **0** | **0** |
| **22** | **Experimental** | **12 Months** | **2** | **0** | **1** | **0** | **1** |
| **22** | **Control** | **1 Month** | **3** | **1** | **1** | **0** | **1** |
| **22** | **Control** | **3 Months** | **7** | **1** | **2** | **1** | **3** |
| **22** | **Control** | **6 Months** | **5** | **1** | **1** | **1** | **2** |
| **22** | **Control** | **12 Months** | **8** | **1** | **1** | **3** | **3** |
| **23** | **Experimental** | **1 Month** | **2** | **0** | **1** | **1** | **0** |
| **23** | **Experimental** | **3 Months** | **1** | **0** | **1** | **0** | **0** |
| **23** | **Experimental** | **6 Months** | **2** | **0** | **1** | **0** | **1** |
| **23** | **Experimental** | **12 Months** | **2** | **0** | **0** | **1** | **1** |
| **23** | **Control** | **1 Month** | **3** | **0** | **1** | **1** | **1** |
| **23** | **Control** | **3 Months** | **4** | **1** | **1** | **1** | **1** |
| **23** | **Control** | **6 Months** | **7** | **1** | **1** | **2** | **3** |
| **23** | **Control** | **12 Months** | **3** | **0** | **1** | **1** | **1** |
| **24** | **Experimental** | **1 Month** | **1** | **0** | **1** | **0** | **0** |
| **24** | **Experimental** | **3 Months** | **3** | **1** | **1** | **0** | **1** |
| **24** | **Experimental** | **6 Months** | **3** | **1** | **1** | **0** | **1** |
| **24** | **Experimental** | **12 Months** | **2** | **0** | **1** | **0** | **1** |
| **24** | **Control** | **1 Month** | **2** | **0** | **1** | **0** | **1** |
| **24** | **Control** | **3 Months** | **5** | **1** | **1** | **1** | **2** |
| **24** | **Control** | **6 Months** | **10** | **1** | **2** | **3** | **4** |
| **24** | **Control** | **12 Months** | **6** | **1** | **1** | **2** | **2** |
| **25** | **Experimental** | **1 Month** | **1** | **0** | **1** | **0** | **0** |
| **25** | **Experimental** | **3 Months** | **3** | **1** | **1** | **0** | **1** |
| **25** | **Experimental** | **6 Months** | **2** | **0** | **1** | **0** | **1** |
| **25** | **Experimental** | **12 Months** | **2** | **0** | **1** | **0** | **1** |
| **25** | **Control** | **1 Month** | **2** | **0** | **1** | **0** | **1** |
| **25** | **Control** | **3 Months** | **5** | **1** | **1** | **1** | **2** |
| **25** | **Control** | **6 Months** | **6** | **1** | **1** | **2** | **2** |
| **25** | **Control** | **12 Months** | **4** | **1** | **0** | **1** | **2** |
| **26** | **Experimental** | **1 Month** | **2** | **0** | **1** | **0** | **1** |
| **26** | **Experimental** | **3 Months** | **2** | **0** | **1** | **0** | **1** |
| **26** | **Experimental** | **6 Months** | **3** | **1** | **1** | **0** | **1** |
| **26** | **Experimental** | **12 Months** | **2** | **0** | **1** | **0** | **1** |
| **26** | **Control** | **1 Month** | **3** | **1** | **1** | **0** | **1** |
| **26** | **Control** | **3 Months** | **6** | **1** | **2** | **1** | **2** |
| **26** | **Control** | **6 Months** | **6** | **1** | **1** | **2** | **2** |
| **26** | **Control** | **12 Months** | **4** | **0** | **1** | **1** | **2** |
| **27** | **Experimental** | **1 Month** | **1** | **0** | **1** | **0** | **0** |
| **27** | **Experimental** | **3 Months** | **4** | **1** | **1** | **1** | **1** |
| **27** | **Experimental** | **6 Months** | **3** | **1** | **1** | **0** | **1** |
| **27** | **Experimental** | **12 Months** | **2** | **0** | **1** | **0** | **1** |
| **27** | **Control** | **1 Month** | **1** | **0** | **1** | **0** | **0** |
| **27** | **Control** | **3 Months** | **3** | **1** | **1** | **0** | **1** |
| **27** | **Control** | **6 Months** | **5** | **1** | **1** | **1** | **2** |
| **27** | **Control** | **12 Months** | **4** | **1** | **0** | **1** | **2** |
| **28** | **Experimental** | **1 Month** | **2** | **0** | **1** | **0** | **1** |
| **28** | **Experimental** | **3 Months** | **2** | **0** | **1** | **0** | **1** |
| **28** | **Experimental** | **6 Months** | **2** | **0** | **1** | **0** | **1** |
| **28** | **Experimental** | **12 Months** | **2** | **0** | **1** | **0** | **1** |
| **28** | **Control** | **1 Month** | **1** | **0** | **1** | **0** | **0** |
| **28** | **Control** | **3 Months** | **5** | **1** | **1** | **1** | **2** |
| **28** | **Control** | **6 Months** | **5** | **1** | **1** | **1** | **2** |
| **28** | **Control** | **12 Months** | **4** | **1** | **0** | **1** | **2** |
| **29** | **Experimental** | **1 Month** | **1** | **0** | **0** | **1** | **0** |
| **29** | **Experimental** | **3 Months** | **3** | **1** | **1** | **1** | **0** |
| **29** | **Experimental** | **6 Months** | **2** | **1** | **1** | **0** | **0** |
| **29** | **Experimental** | **12 Months** | **2** | **0** | **1** | **0** | **1** |
| **29** | **Control** | **1 Month** | **2** | **0** | **1** | **0** | **1** |
| **29** | **Control** | **3 Months** | **6** | **1** | **2** | **1** | **2** |
| **29** | **Control** | **6 Months** | **7** | **1** | **1** | **2** | **3** |
| **29** | **Control** | **12 Months** | **5** | **1** | **1** | **1** | **2** |
| **30** | **Experimental** | **1 Month** | **1** | **0** | **1** | **0** | **0** |
| **30** | **Experimental** | **3 Months** | **3** | **1** | **1** | **1** | **0** |
| **30** | **Experimental** | **6 Months** | **2** | **0** | **1** | **0** | **1** |
| **30** | **Experimental** | **12 Months** | **2** | **0** | **1** | **0** | **1** |
| **30** | **Control** | **1 Month** | **3** | **1** | **1** | **0** | **1** |
| **30** | **Control** | **3 Months** | **1** | **0** | **1** | **0** | **0** |
| **30** | **Control** | **6 Months** | **5** | **1** | **1** | **1** | **2** |
| **30** | **Control** | **12 Months** | **4** | **1** | **0** | **1** | **2** |
| **31** | **Experimental** | **1 Month** | **1** | **0** | **1** | **0** | **0** |
| **31** | **Experimental** | **3 Months** | **4** | **1** | **1** | **1** | **1** |
| **31** | **Experimental** | **6 Months** | **2** | **0** | **1** | **0** | **1** |
| **31** | **Experimental** | **12 Months** | **1** | **0** | **0** | **0** | **1** |
| **31** | **Control** | **1 Month** | **1** | **0** | **1** | **0** | **0** |
| **31** | **Control** | **3 Months** | **2** | **0** | **1** | **0** | **1** |
| **31** | **Control** | **6 Months** | **6** | **1** | **1** | **2** | **2** |
| **31** | **Control** | **12 Months** | **5** | **1** | **1** | **1** | **2** |
| **32** | **Experimental** | **1 Month** | **3** | **1** | **1** | **1** | **0** |
| **32** | **Experimental** | **3 Months** | **2** | **0** | **1** | **0** | **1** |
| **32** | **Experimental** | **6 Months** | **3** | **1** | **1** | **0** | **1** |
| **32** | **Experimental** | **12 Months** | **2** | **0** | **0** | **1** | **1** |
| **32** | **Control** | **1 Month** | **5** | **1** | **2** | **0** | **2** |
| **32** | **Control** | **3 Months** | **1** | **0** | **1** | **0** | **0** |
| **32** | **Control** | **6 Months** | **5** | **1** | **1** | **1** | **2** |
| **32** | **Control** | **12 Months** | **4** | **0** | **1** | **1** | **2** |
| **33** | **Experimental** | **1 Month** | **2** | **0** | **1** | **0** | **1** |
| **33** | **Experimental** | **3 Months** | **4** | **1** | **1** | **1** | **1** |
| **33** | **Experimental** | **6 Months** | **3** | **1** | **1** | **0** | **1** |
| **33** | **Experimental** | **12 Months** | **2** | **0** | **1** | **0** | **1** |
| **33** | **Control** | **1 Month** | **4** | **1** | **1** | **1** | **1** |
| **33** | **Control** | **3 Months** | **3** | **1** | **1** | **0** | **1** |
| **33** | **Control** | **6 Months** | **7** | **1** | **1** | **2** | **3** |
| **33** | **Control** | **12 Months** | **5** | **1** | **1** | **1** | **2** |
| **34** | **Experimental** | **1 Month** | **1** | **0** | **1** | **0** | **0** |
| **34** | **Experimental** | **3 Months** | **3** | **1** | **1** | **0** | **1** |
| **34** | **Experimental** | **6 Months** | **2** | **1** | **1** | **0** | **0** |
| **34** | **Experimental** | **12 Months** | **3** | **1** | **1** | **0** | **1** |
| **34** | **Control** | **1 Month** | **3** | **1** | **1** | **0** | **1** |
| **34** | **Control** | **3 Months** | **5** | **1** | **1** | **1** | **2** |
| **34** | **Control** | **6 Months** | **6** | **1** | **1** | **2** | **2** |
| **34** | **Control** | **12 Months** | **4** | **1** | **0** | **1** | **2** |
| **35** | **Experimental** | **1 Month** | **2** | **0** | **1** | **0** | **1** |
| **35** | **Experimental** | **3 Months** | **3** | **1** | **1** | **0** | **1** |
| **35** | **Experimental** | **6 Months** | **3** | **1** | **1** | **0** | **1** |
| **35** | **Experimental** | **12 Months** | **2** | **0** | **1** | **0** | **1** |
| **35** | **Control** | **1 Month** | **3** | **1** | **1** | **0** | **1** |
| **35** | **Control** | **3 Months** | **6** | **1** | **2** | **1** | **2** |
| **35** | **Control** | **6 Months** | **7** | **1** | **1** | **2** | **3** |
| **35** | **Control** | **12 Months** | **4** | **1** | **1** | **1** | **1** |
| **36** | **Experimental** | **1 Month** | **1** | **0** | **1** | **0** | **0** |
| **36** | **Experimental** | **3 Months** | **3** | **1** | **1** | **0** | **1** |
| **36** | **Experimental** | **6 Months** | **3** | **1** | **1** | **0** | **1** |
| **36** | **Experimental** | **12 Months** | **2** | **0** | **1** | **0** | **1** |
| **36** | **Control** | **1 Month** | **1** | **0** | **1** | **0** | **0** |
| **36** | **Control** | **3 Months** | **4** | **1** | **1** | **1** | **1** |
| **36** | **Control** | **6 Months** | **5** | **1** | **1** | **1** | **2** |
| **36** | **Control** | **12 Months** | **6** | **1** | **1** | **2** | **2** |
| **37** | **Experimental** | **1 Month** | **2** | **0** | **1** | **1** | **0** |
| **37** | **Experimental** | **3 Months** | **3** | **1** | **1** | **0** | **1** |
| **37** | **Experimental** | **6 Months** | **2** | **1** | **1** | **0** | **0** |
| **37** | **Experimental** | **12 Months** | **2** | **0** | **1** | **0** | **1** |
| **37** | **Control** | **1 Month** | **3** | **1** | **1** | **0** | **1** |
| **37** | **Control** | **3 Months** | **6** | **1** | **2** | **1** | **2** |
| **37** | **Control** | **6 Months** | **6** | **1** | **1** | **2** | **2** |
| **37** | **Control** | **12 Months** | **6** | **1** | **1** | **2** | **2** |
| **38** | **Experimental** | **1 Month** | **1** | **0** | **1** | **0** | **0** |
| **38** | **Experimental** | **3 Months** | **3** | **1** | **1** | **1** | **0** |
| **38** | **Experimental** | **6 Months** | **3** | **1** | **1** | **0** | **1** |
| **38** | **Experimental** | **12 Months** | **1** | **0** | **0** | **0** | **1** |
| **38** | **Control** | **1 Month** | **3** | **0** | **2** | **0** | **1** |
| **38** | **Control** | **3 Months** | **2** | **0** | **1** | **0** | **1** |
| **38** | **Control** | **6 Months** | **5** | **1** | **1** | **1** | **2** |
| **38** | **Control** | **12 Months** | **4** | **0** | **1** | **1** | **2** |
| **39** | **Experimental** | **1 Month** | **1** | **0** | **1** | **0** | **0** |
| **39** | **Experimental** | **3 Months** | **3** | **1** | **1** | **0** | **1** |
| **39** | **Experimental** | **6 Months** | **3** | **1** | **1** | **0** | **1** |
| **39** | **Experimental** | **12 Months** | **3** | **1** | **1** | **0** | **1** |
| **39** | **Control** | **1 Month** | **3** | **1** | **1** | **0** | **1** |
| **39** | **Control** | **3 Months** | **2** | **0** | **1** | **0** | **1** |
| **39** | **Control** | **6 Months** | **5** | **1** | **1** | **1** | **2** |
| **39** | **Control** | **12 Months** | **5** | **1** | **1** | **1** | **2** |
| **40** | **Experimental** | **1 Month** | **2** | **0** | **1** | **0** | **1** |
| **40** | **Experimental** | **3 Months** | **3** | **1** | **1** | **0** | **1** |
| **40** | **Experimental** | **6 Months** | **2** | **0** | **1** | **0** | **1** |
| **40** | **Experimental** | **12 Months** | **2** | **0** | **1** | **0** | **1** |
| **40** | **Control** | **1 Month** | **2** | **0** | **1** | **0** | **1** |
| **40** | **Control** | **3 Months** | **4** | **1** | **1** | **1** | **1** |
| **40** | **Control** | **6 Months** | **6** | **1** | **1** | **2** | **2** |
| **40** | **Control** | **12 Months** | **6** | **1** | **1** | **2** | **2** |
